# Design and Validation of Experimental Setup for Cell Spheroid Radiofrequency-Induced Heating

**DOI:** 10.3390/s23094514

**Published:** 2023-05-05

**Authors:** Ioannis Androulakis, Riccardo Ferrero, Rogier van Oossanen, Alessandra Manzin, Antonia G. Denkova, Kristina Djanashvili, Robin Nadar, Gerard C. van Rhoon

**Affiliations:** 1 Department of Radiotherapy, Erasmus MC Cancer Institute, University Medical Center, 3015 GD Rotterdam, The Netherlands; i.androulakis@erasmusmc.nl (I.A.);; 2 Istituto Nazionale di Ricerca Metrologica (INRIM), 10135 Turin, Italy; 3 Department of Radiation Science and Technology, TU Delft, 2629 JB Delft, The Netherlands; 4 Department of Biotechnology, TU Delft, 2629 HZ Delft, The Netherlands

**Keywords:** hyperthermia, induced, cells, cultured, tumor cells, cultured, drug screening assays, antitumor, combined modality therapy, electromagnetic fields

## Abstract

While hyperthermia has been shown to induce a variety of cytotoxic and sensitizing effects on cancer tissues, the thermal dose–effect relationship is still not well quantified, and it is still unclear how it can be optimally combined with other treatment modalities. Additionally, it is speculated that different methods of applying hyperthermia, such as water bath heating or electromagnetic energy, may have an effect on the resulting biological mechanisms involved in cell death or in sensitizing tumor cells to other oncological treatments. In order to further quantify and characterize hyperthermia treatments on a cellular level, in vitro experiments shifted towards the use of 3D cell spheroids. These are in fact considered a more representative model of the cell environment when compared to 2D cell cultures. In order to perform radiofrequency (RF)-induced heating in vitro, we have recently developed a dedicated electromagnetic field applicator. In this study, using this applicator, we designed and validated an experimental setup which can heat 3D cell spheroids in a conical polypropylene vial, thus providing a reliable instrument for investigating hyperthermia effects at the cellular scale.

## 1. Introduction

The use of heat as a single therapeutic agent or in combination with other treatments goes back centuries [1]. The positive results reported in patients treated with hyperthermic, i.e., at 39–44 °C, regional isolated perfusion in limbs in 1967 by Cavaliere et al. [2] boosted the interest in hyperthermia. Extensive experimental research was performed to investigate the biological mechanisms that contribute to the increased effectiveness of cell killing when radiotherapy and chemotherapy are combined with hyperthermia [3]. Initially, the experimental biological research focused on the cytotoxic effects of hyperthermia, which resulted in challenging requirements for the goal of clinical hyperthermia [4], i.e., achieving tumor temperatures > 42 °C for 1 h or more. Follow-up biological research focused on the broader biological effects of hyperthermia. The review by Dewhirst et al. provided a retrospective analysis of how the early experimental studies from the late 1970s and the early 1980s on hyperthermia biology put a lot of emphasis on the necessity of killing cells with a single hyperthermia treatment to be considered successful [5], thereby strongly influencing the clinical application of hyperthermia. New in vitro and in vivo biological research from the late 1980s and onwards demonstrated that hyperthermia induced a plethora of biological effects, each with its optimal temperature interval [6,7]. As a consequence of the Dewhirst et al. review, the requirement on the applied thermal dose during clinical hyperthermia has become more realistic and modest in the homogeneity and peak of the temperature, whereas the duration of the clinical treatment has remained between 60 and 90 min depending on whether the heating is superficial or deep. Still, the definitions of the optimal thermal dose, as well as the optimal duration between the two modalities, radiotherapy or chemotherapy, and hyperthermia, are not settled and are the subject of ongoing discussions [8,9,10].

An interesting and so-far not entirely addressed question is whether the different methods of applying hyperthermia affect the resulting biological mechanism involved in direct cell killing or in tumor sensitization to therapies. In other words, does hyperthermia via water bath heating for 1 h at 42 °C cause the same effect as when the same thermal dose is applied using electromagnetic (EM) or ultrasound energy? The existing literature points to the complexity of the answer to this question. Various groups have reported a difference in the sensitizing effect of hyperthermia when heating is applied either via EM energy or through thermal conduction using a hot water bath [11]. Tumor-reducing effects are suggested to be induced specifically by EM exposure during the EM-based hyperthermia of tumor cells. The most well-known is modulated electro-hyperthermia (mEHT)—a product of Oncotherm (Oncotherm Kft, Budaörs, Hungary). As reported by the company, the principles of mEHT are based on classical hyperthermia, but the aim, besides the absolute increase in temperature, is direct electric-field energy absorption in the extracellular liquid and the destruction of the membrane of the cancer cells. By using a time-fractal mEHT, with a carrier frequency of 13.56 MHz, the selective heating of cell membrane rafts is claimed to be achieved. This selective heating has been linked to cellular changes in the targeted cells via thermal and non-thermal mechanisms [12]. Overall, in experimental studies, it is reported that mEHT can lead to enhanced cell apoptosis compared to water bath heating [12].

Recent research by the hyperthermia group at the Charité—Universitätsmedizin Berlin demonstrated that very low-frequency amplitude-modulated radiofrequency hyperthermia (VLF-AM-RF-HT) cannot induce relevant temperature-dependent effects at the cell membrane ducts [13]. However, additional research of the group suggests that VLF-AM-RF-HT promotes membrane vibrations at specific resonance frequencies. The latter may indicate that non-thermal antiproliferative membrane effects occur via ion disequilibrium and/or resonances causing membrane depolarization, the opening of certain channels, or even hole formation. In addition, they conclude that the effect of VLF-AM-RF-HT may be tumor-specific owing to cancer-specific ion channels and because, with increasing malignancy, membrane elasticity parameters may differ from those in normal tissues [14,15].

The existence of tumor-specific frequencies in the very low-frequency range has also been reported by others [16,17]. In 2004 Kirson et al. reported that when properly tuned, very low-intensity, intermediate-frequency (100–300 kHz) electric fields, called tumor treatment fields (TTF), stunt the growth of cancerous cells [18]. The non-thermal mechanism responsible for the interruption of rapid cell division, thus tumor growth, is explained by Kirson et al. [19]. Although the TTF device has been approved by the Food and Drug Administration (FDA) for the treatment of glioblastoma, there is still ongoing discussion on the recognition of the TTF as a standard of care [20]. A difference in response to the heat exposure using higher-frequency EM fields without low-frequency amplitude modulation or using water bath heating has been reported as well. In particular, a study by Hader et al. suggests that non-thermal factors contribute to breast cancer cell killing (in vitro) and also shape the immunogenic properties of the tumor cells when using microwave heating instead of water bath heating [21].

In light of the above-reported differences in treatment effectiveness noted between water bath and EM heating, the metrological community started the Project 18HLT06 RaCHy “Radiotherapy Coupled with Hyperthermia”, in the framework of the European Metrology Programme for Innovation and Research (EMPIR), to investigate the biological differences between the various heating systems using 3D tumor cell spheroid cultures [22]. The outcome of this study is important for the quantification of the increased cell killing introduced when adding hyperthermia to radiotherapy and to translate the result to a biological equivalent dose.

The introduction of 3D tumor cell spheroids provides an in vitro model that more closely resembles in vivo tumor cell behavior, and hence might lead to a better model for hyperthermia-induced radiosensitization [23]. Assessing the behavior of tumor cells using traditional two-dimensional (2D) cell culture methods poses challenges in accurately reflecting the in vivo cell microenvironment [24]. One of the limitations of 2D culture is its inability to fully replicate the complex cell–matrix and cell–cell interactions that contribute to tissue or tumor growth. To address this issue, researchers have developed alternative approaches that incorporate extracellular matrix components to create more physiologically representative in vitro environments. However, these methods often only allow for the assessment of individual cell behavior, failing to capture the complexity of in vivo interactions. A more recent approach that better replicates the compact architecture of in vivo cell growth is the use of multicellular spheroids. Spheroids can be utilized to investigate cell–matrix interactions of normal cells but also serve as tumor analogs to model tumor progression characteristics such as metastatic growth or drug resistance [25]. Spheroids can be formed through the proliferation of single cells embedded in a matrix or through the aggregation of multiple cells into a single cell cluster using methods such as hanging drop or centrifugation. The use of spheroids offers a biologically relevant and versatile platform for studying tumor cell behavior in a more physiologically relevant environment. However, the ability to assess therapeutic responses in spheroids is constrained by the scarcity of handling technologies/equipment and the absence of simple and cost-effective standardized assays, both of which pose limitations to the practical application of this approach, as reviewed by Costa et al. [26].

In the past, we designed and characterized, in phantoms, an RF applicator for the controlled and focused in vitro heating of tumor cells using EM energy [27]. In this current study, we propose and validate an experimental setup with the proposed RF applicator for the RF-induced heating of 3D tumor cell spheroids in a polypropylene (PP) vial.

## 2. Materials and Methods

### 2.1. Cell Cultures

U87 MG (malignant glioma) cell lines derived from human malignant glioblastoma were obtained from the VU University Medical Center Amsterdam. The liquid-overlay method was used to obtain spheroids by seeding the cells in cell-repellent, U-shaped 96-well plates (CELLSTAR^®^, Greiner Bio-One GmbH, Kremsmünster, Austria), at a concentration of 2000 cells per 200 μL medium [28]. The 3D U87 spheroids were prepared using a culture medium containing high-glucose (4.5 g/L) Dulbecco’s Modified Eagle’s medium, supplemented with 10% fetal bovine serum (Biowest, Nuaillé, France) and 1% combined 6 mg/L penicillin and 10 mg/L streptomycin antibiotics (Biowest, Nuaillé, France). Following the seeding of the cancer cells, the plate was incubated and left untouched for the first three days to ensure the initial formation of individual spheroids in each of the 96-well plates. After the initial 7 days, half of the medium (100 μL) was replaced with new medium to ensure that the spheroids did not undergo nutrient limitation. Seven days after the initial seeding of the spheroids, half of the medium was replaced with fresh medium every three to four days.

### 2.2. Spheroid Volume and Viability Analysis

Spheroids were imaged using a 12-megapixel camera mounted on the binocular microscope using automated imaging software (SampleScan, SPT Labtech Ltd., Melbourn, UK). The images were analyzed using the open-source image processing software, ImageJ (v1.5, NIH, Bethesda, MD, USA). The volume was determined based on the 2D area measurement, with the assumption that the spheroid was a perfect sphere. However, it should be noted that the spheroidal shape was affected post-treatment, which led to a large standard deviation.

A CellTiter-Glor 3D cell viability assay (REFG9682) was performed to determine the therapeutic effect of heat treatment. The U87 spheroids’ viability was measured on day 7 after heat treatment exposure. A Glo3D reagent and culture medium mixture was prepared with a volume ratio of 1:1. The medium surrounding the spheroids was carefully removed, and 200 μL of the Glo3D reagent in culture medium was added per well. After every 10 min, the contents of the wells were mixed by pipetting up and down to ensure thorough mixing. A light microscope was used to confirm the complete spheroid dissociation after 40 min. Finally, the luminescence of each well was measured using a fluorescence spectrophotometer. The setup parameters of the fluorescence spectrophotometer included: bio/chemo-luminescence data mode; emission wavelength of 600 nm; emission slit of 20 nm; open emission filter; gate time of 200 ms [29].

All statistical analyses were performed using Prism (v8.4.0, GraphPad Software Inc., La Jolla, CA, USA). All the results were presented as the mean ± the standard deviation, where the mean is an average of at least 3 independent repeats and the number of samples in each group is 6. *p*-values were determined using the one-way or two-way ANOVA followed by the Bonferroni test. A *p*-value of *p* < 0.05 was assumed to indicate significantly different results.

### 2.3. Heating Target

**Figure 1:**
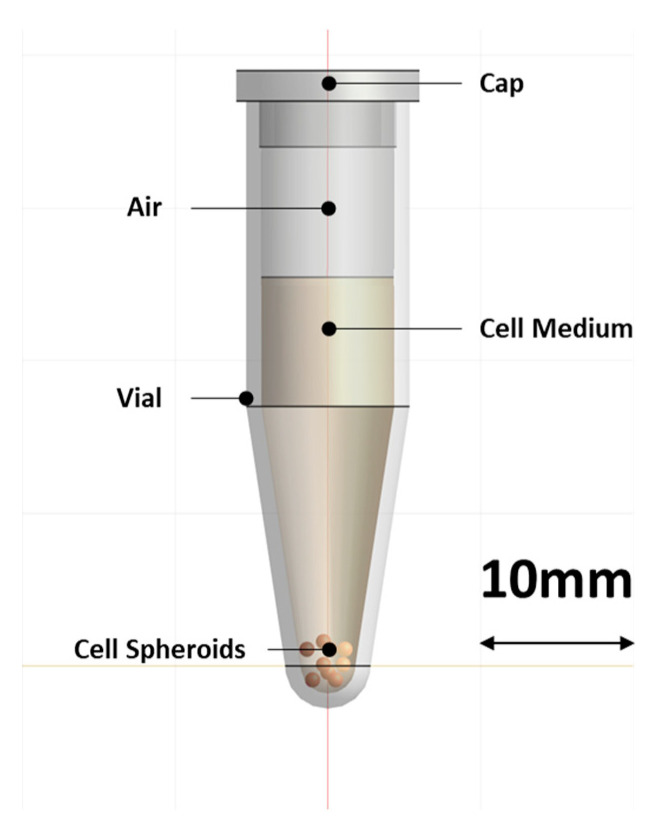
Schematic of the conical PP vial with the cell medium and the position of the 3D cell spheroids, as it was used in the simulation model.

Each spheroid had an average diameter of 346.9 ± 12 µm. For each heating experiment, six spheroids were placed in a conical polypropylene (PP) screw-top Eppendorf vial (1.5 mL). Each vial was partially filled with 1 mL of the same cell medium, leaving 0.5 mL of air in the top portion, and incubated at 37 °C in an incubator until they were ready for heat treatment. During this time, the spheroids settled to the bottom of the vial (Figure 1). Prior to heat treatment, the PP vial was transferred out of the incubator. After heat treatment, the spheroids were slowly separated with the help of a 1 mL micropipette by tilting the 1.5 mL Eppendorf tube at 45° and slowly aspirating them, using a 1 mL micropipette tip with the tip cut to an opening larger than 1000 µm to ensure the spheroids were not disturbed/dissociated while transferring them back to their individual wells. To ensure this separation did not affect the spheroids, their sizes were measured before and after treatment, but the change in size was found to be insignificant and constant throughout the treatment groups, including the control group.

### 2.4. EM Hyperthermia Applicator Setup

**Figure 2:**
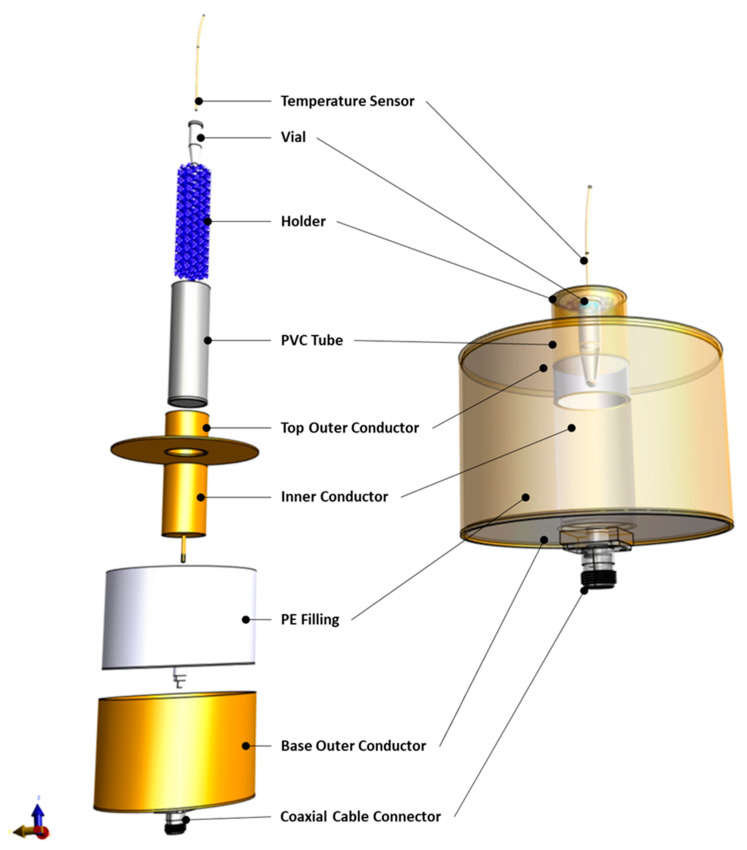
Model of the RF applicator components separately (**left**) as well as assembled (**right**).

The RF applicator that was used in this study is a modified transverse EM-mode (TEM) applicator according to an earlier design by Lagendijk [30], which was redesigned for in vitro studies and potentially for preclinical studies on small animals. The applicator consists of an outer cylindrical conductor connected to the metallic shield of the feeding coaxial cable and an inner hollow cylindrical conductor connected to the coaxial cable core. Its design ensures the generation of an EM field in a targeted region at the center of the aperture, where a sample, housed in a polyvinyl chloride (PVC) tube, can be positioned. The EM field is circumferential and radiative, with a heating focus at the center of the aperture. The space between the two conductors is filled with a dielectric medium made of polyethylene. A more comprehensive description of the applicator design (Figure 2) can be found in [27].

To position the conical heating target, coarsely reticulated polyether foam (10–20 ppi) was used as a target holder. The foam was cut to shape to hold the heating target with its bottom part in the center of the heating focus of the applicator (Figure 2). The PVC tube containing the holder and the heating target was filled with a saline solution, which served as a matching medium between the applicator and the heating target, and its salinity was adjusted to increase the efficiency of the applicator. For the current setup, EM simulations of the applicator with adapted electrical conductivities were performed. The salinity that gives the lowest Voltage Standing Wave Ratio (VSWR), i.e., the lowest reflected power in the transmission line, corresponds to the optimal electrical conductivity of the matching medium, resulting in the highest efficiency of energy transfer to the vial.

**Figure 3:**
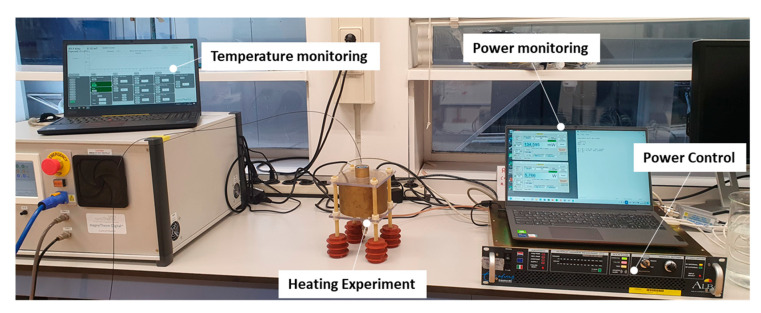
The experimental setup. Temperature monitoring is performed through a fiberoptic temperature sensor inserted in the PP vial. The applicator power is controlled through a 434 MHz power amplifier and monitored through power meters connected through a bidirectional coupler to the RF power output.

The complete experimental setup can be seen in Figure 3. To monitor the temperature increase achieved in the heating target, a PRB-G40-2.0M-STM-MRI fiber optic temperature sensor (Osensa Innovation, Burnaby, BC, Canada) was used. The fiberoptic temperature sensor was sterlized with 70% ethanol to ensure sterility before being used. After that, it was inserted in the vial through a hole in the center of the cap and positioned with its measuring tip on the bottom of the vial. The applicator was powered by a custom 200 W 434 MHz RF power generator (Medlogix, Rome, Italy) through a 50 Ω coaxial cable. The forward and reflected power was monitored using two PWR-4GHS power meters by Mini Circuits (Brooklyn, NY, USA), connected through a −50dB bidirectional coupler.

### 2.5. EM Hyperthermia Experimental Procedure

For each heating experiment, a predefined heating time (*t*_target_) and temperature (*T*_target_) were set, and the cummulative equivalent minutes at the 43 °C parameter (CEM43) was calculated according to the following formula:

(1) $$\mathrm{CEM}43=t_{\mathrm{target}}R^{43-T_{\mathrm{target}}}$$

where

(2) $$\left\{\begin{array}{c} R=0,\;\;T\leq 37\;{}^{\circ}C\;\\ R=0.25,\;\;37<T\leq 43\\ R=0.5,\;\;T>43\;{}^{\circ}C \end{array}\right.{}^{\circ}C$$

and where *t*_target_ is specified in minutes.

The PP vial containing the spheroids was rapidly transferred into the applicator and accurately positioned thanks to the mesh holder. The temperature probe was inserted in the sample, and temperature measurements were initiated. Initially, high power was applied to rapidly increase the temperature in the target; then, the power was gradually reduced until a stable temperature *T*_target_ was reached. The power adaptation was performed manually, while monitoring the temperature probe measurement. The constant temperature *T*_target_ was kept for a period equal to *t*_target_. After that, the power was turned off and the sample was removed and returned to the incubator.

The actual CEM43 parameter was recalculated based on the real temperature exposure according to:

(3) $$\mathrm{CEM}43=\overset{K}{\underset{k=1}{\sum }}\Delta t_{k}R^{43-T\left(t_{k}\right)}$$

where the interval $\left[0,t_{\mathrm{target}}\right]$ is sampled in *K* intervals and $\Delta t_{k}=t_{k}-t_{k-1}$ corresponds to the thermometer sampling resolution of 1 s.

### 2.6. Material Properties

The heating target in this study consisted of a conical PP vial and a cell medium solution containing cells. The EM properties of the PP material and saline solution were estimated based on values in the literature [27,31,32], while the EM properties of the cell medium were measured using a Dielectric Assessment Kit (DAK) 12 (4 MHz–3 GHz) probe (SPEAG, Zurich, Switzerland). The thermal properties of the cell medium were based on the thermal properties of blood [33]. The density values of the materials are taken from the literature. The EM and thermal properties used in this study are summarized in Table 1.

### 2.7. Simulation of Heating Experiment

All the simulations were performed using the computation platform Sim4Life (v6.2, Zurich Med Tech, Zurich, Switzerland). For the EM simulations, a full-wave Finite-Difference Time-Domain (FDTD) solver of Maxwell’s equations was used. An edge source with an impedance of 50 Ω and emitting a monochromatic harmonic wave of 434 MHz was employed to power the applicator. All metallic parts of the applicator were modeled as perfect electric conductors, while all other materials were simulated as dielectric materials, using the properties reported in Table 1. The computational domain comprised an empty air box encasing the whole applicator. The box sides were at a distance of 173.6 mm from the applicator in every direction. The heating target and applicator were modeled as illustrated in Figure 1 and Figure 2, respectively. An absorbing boundary condition was used for the boundaries of the computational domain. The VSWR and specific absorption rate (SAR) distribution were computed using the built-in post-processing pipeline of Sim4Life.

For the temperature simulations, a transient FDTD solver of the Pennes’ equation was used, considering the power loss density calculated with the EM simulation as the input heating source. All metallic parts of the applicator were ignored, while, for the other materials, the properties were assigned according to Table 1. A Dirichlet boundary condition with a constant temperature equal to room temperature was used as a boundary condition, which was assumed at the outer surface of the applicator.

## 3. Results

### 3.1. Optimal Saline Solution

**Figure 4:**
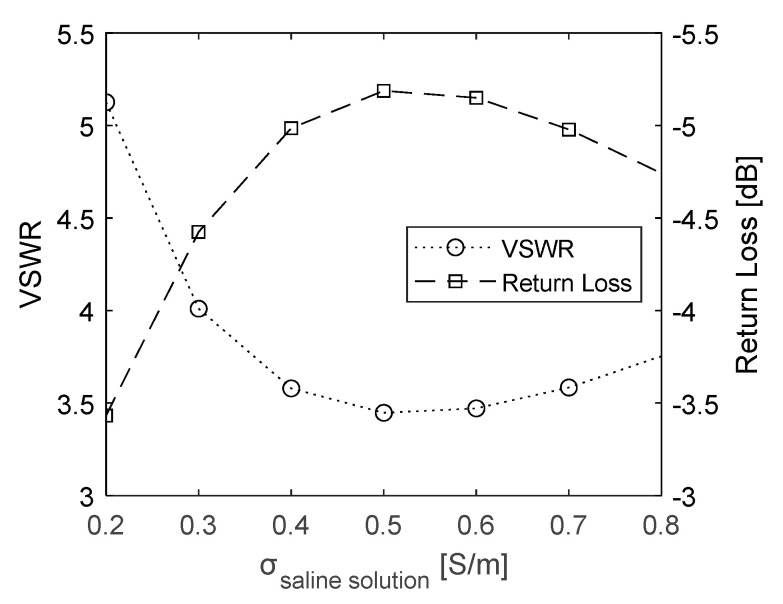
VSWR and Return Loss values calculated as a function of saline solution conductivity.

To evaluate the optimal salinity of the matching medium between the outer wall of the conical heating target and the applicator, electrical conductivity levels between 0.2 and 0.8 S/m were considered. EM simulations were performed to compute the effect of conductivity on the efficiency of the transmission of the RF power through the transmission line and the power of the signal reflected by discontinuities in the transmission line. Figure 4 shows the VSWR and Return Loss resulting from the simulations, as a function of the saline solution conductivity. The optimal conductivity corresponding to the lowest value of VSWR and the highest value of Return Loss is around 0.5 S/m. This target value can be achieved by adding 2.6 g of NaCl per liter of deionized water [31,32]. Therefore, this saline solution was used for the heating experiments.

### 3.2. Predicted SAR and Temperature Distribution

**Figure 5:**
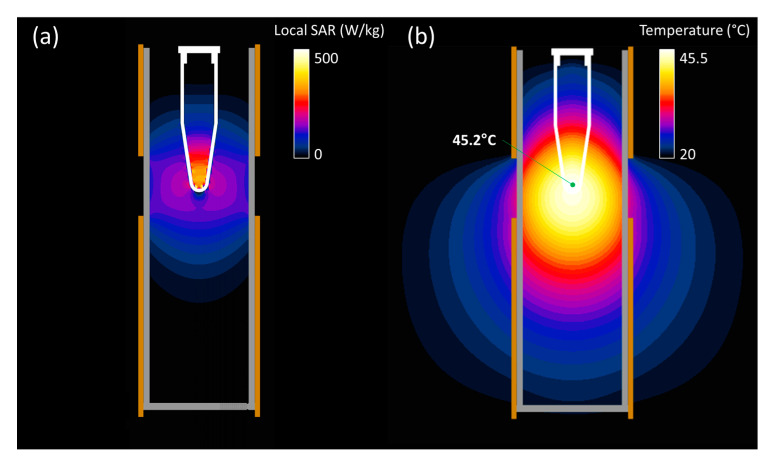
Simulated local SAR (**a**) and steady-state temperature (**b**) spatial distribution in the central cross-section of the RF applicator when applying 3 W of effective power. The temperature reached at the measuring point is indicated in the latter plot.

The absorption of power that led to heat mainly took place in the apex of the vial. This can be seen in Figure 5a, where the local SAR distribution is shown. The steady-state temperature distribution resulted in a wider area achieving hyperthermia temperatures, but only with the vial apex reaching the maximum temperature of 45.5 °C. This setup ensured that the heating was the result of direct EM power application, instead of conducted heat.

### 3.3. Thermal Dose Reproducibility

**Figure 6:**
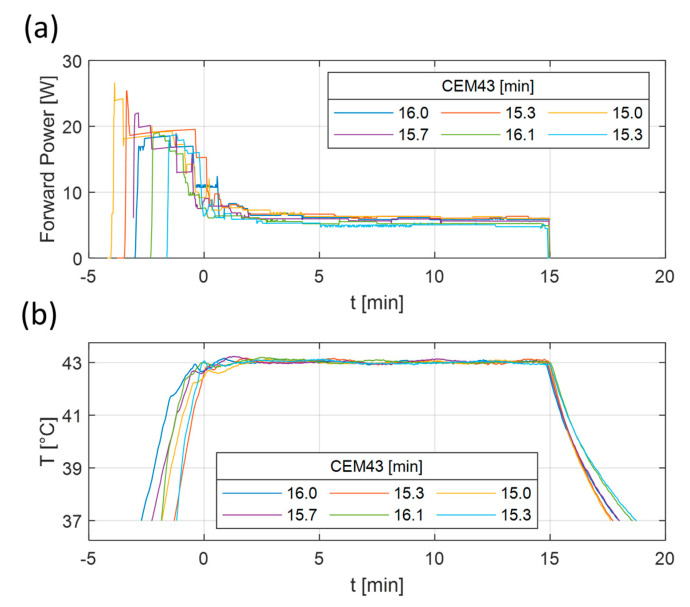
Measured forward power (**a**) and temperature (**b**) results of six repeated heating experiments. The heating experiments were planned to deliver 43 °C for 15 min. The lines correspond to the measured temperature distribution at the tip of the vial. The corresponding CEM43 values of each experiment are visible in the legend.

To investigate the reproducibility of the heating experiments, a series of tests using a CEM43 dose of 15 min (43 °C for 15 min) were repeated six times on vials containing the cell medium, but without the spheroids. The power and temperature levels of the repeated experiments can be seen in Figure 6. The resulting CEM43 had a value of 15.6 ± 0.4 min (mean ± std), or 103.7 ± 2.9% of the original intended dose. It can be seen in Figure 6b that the cool down time after turning off the power contributed to the total thermal dose. Therefore, it was decided that the heating target would be removed from the applicator immediately after the power was turned off to increase the cooling down speed.

### 3.4. Heating Experiment Results

**Figure 7:**
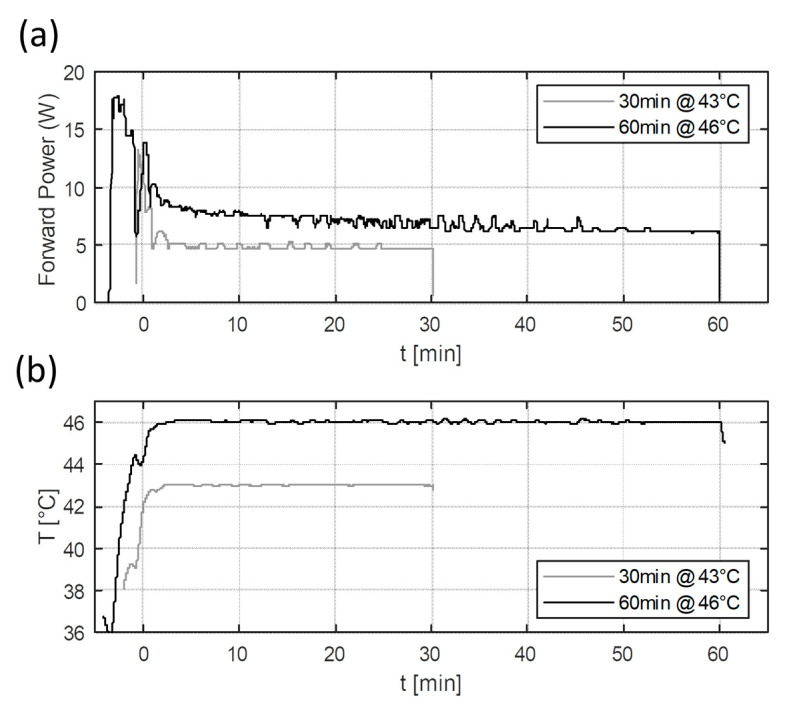
Measured forward power (**a**) and temperature (**b**) results of the two heating experiments. The heating experiments were planned to deliver 43 °C for 30 min and 46 °C for 60 min. The lines correspond to the measured temperature distribution at the tip of the vial.

Figure 7 shows the power levels and temperature reached during the heating experiments. The required temperature could be reached in a few minutes of preheating with high power levels (>10 W), after which, a relatively stable temperature could be maintained using stable lower power levels. In the experiments, the initial targets for CEM43 were 480 min (46 °C for 60 min) and 30 min (43 °C for 30 min). The final reached CEM43 values were 493.6 min (2.8% error) and 30 (0.0% error), respectively. Hence, the intended thermal dose can be met with the current setup with low errors. In the future, adding the continuous monitoring of CEM43 values could allow for more precise thermal dose delivery.

**Figure 8:**
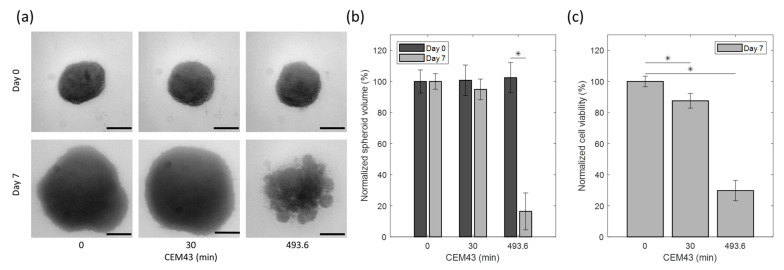
Effect of EM-based heat treatment on U87 spheroids: (**a**) representative spheroid images corresponding to different hyperthermia conditions on day 0 and day 7. Horizontal lines correspond to 200 µm. (**b**) Percentage of spheroid volume normalized to control spheroids on day 0 and day 7 post-heat treatment. (**c**) Percentage of cell viability of spheroids normalized to control spheroids on day 7 post heat treatment. * Statistical significance as determined via two-way ANOVA with a Bonferroni (multiple comparisons) post hoc test.

In Figure 8a, U87 MG human tumor-derived glioblastoma cell line spheroids from the heating experiments reported in Figure 7 are shown before and seven days after heat application. The treatment groups were: control (no thermal dose applied); hyperthermia CEM43 dose of 30 min; ablative CEM43 dose of 493.6 min. The spheroid volume and viability results from each treatment group were averaged and compared between groups. Each treatment group was a result from a single heating experiment. The control group was handled similarly to the other treatment groups with the exception that it was not placed in the RF applicator. As can be seen in Figure 8b, the volume of the spheroids showed a marginal (5.2%) non-significant reduction for CEM43 equal to 30 min (*p*-value > 0.05), which is in agreement with the minimal direct cytotoxicity expected in hyperthermia. However, for the CEM43 dose of 493.6 min, there was a drastic decrease (83.6%) in volume compared to the control (*p*-value < 0.001), which is in agreement with the direct cytotoxic effect expected with ablative thermal doses. A similar response was also observed by measuring the viability of the spheroids after heat treatment, as shown in Figure 8c. The spheroid viability was reduced to 87.5 ± 4.7% with the CEM43 dose of 30 min, whereas the viability reduced remarkably to 29.8 ± 6.5% with the CEM43 dose of 493.6 min. In both treatments, the viability reduction was significant (*p*-value < 0.001).

## 4. Discussion

Hyperthermia is known to have complex cytotoxic as well as radio- and chemosensitizing effects on tumor cells. In general, in current in vitro and in vivo experimental research to investigate the biological effects of hyperthermia, the most dominant way to expose a sample to an increased temperature (i.e., thermal dose) is based on water bath heating.

Our knowledge of the biological effects of hyperthermia has grown strongly over the past few decades, especially concerning molecular radiobiological aspects. There is also a growing understanding that the biological response to heat substantially depends on the tissue type and origin of each tumor cell. So, while understanding of the biological effects is growing, we still have sufficient progress to make towards a well-quantified definition of the thermal dose–effect relationships. The quantification of the cytotoxic and sensitizing effects of a certain thermal dose to a specific type of tumor is important for clinical translation, as it will allow for better treatment design [34]. Furthermore, there is ongoing speculation that additional non-thermal effects might be induced by RF-based hyperthermia. However, the biological mechanisms that are involved and lead to these non-thermal effects are, at this moment, not sufficiently understood and are still the subject of ongoing discussion.

Irrespective of the different biological effects, there is an increasing belief that the use of 3D cell spheroids in in vitro research provides important benefits over studying cells grown as 2D cultures. Overall, there is a common expectation that 3D cell spheroids more closely resemble the in vivo environment of cancerous cells, and therefore, have better potential to improve our understanding of how hyperthermia, and the way it is induced (e.g., RF or US), affects cancer cell response. This might in extension lead to a more quantified documentation of heat-related cell death mechanisms.

To achieve these objectives, we investigated the ability to apply the focused RF-induced heating of 3D cell spheroid cell cultures. To the best of our knowledge, this is the first attempt to develop such an experimental setup, although similar efforts for in vivo experiments have been reported and have the potential to be adapted for in vitro experiments [35]. Similarly to the current effort, a focused US applicator has recently been developed and has the ability to be used for similar heating targets [36]. Hereto, we used our recently developed 434 MHz TEM applicator to prepare an experimental setup for the electromagnetic RF heating of 3D cell spheroids in a PP vial.

The applicator can easily achieve a focused heating region that is geometrically stable and not dependent on interference scenarios, as is the case in phased-array applicators. The fact that the heating focus is geometrically dependent makes the system robust and reliable. Further robustness is ensured by using a saline solution as a matching medium between the applicator and the heating target. The saline solution lowers the power requirements by making the system more efficient and also reduces sharp temperature gradients between the vial and an eventual air layer. Furthermore, by maintaining a lower conductivity in the saline solution compared to the cell medium, power deposition is highest in the target region rather than the surrounding area. Fiber-optical temperature monitoring ensures the real-time monitoring of the temperature in the heating target, without risk of EM interference. Finally, the heat treatment with the current setup is easy to use since the PP vial positioning as well as the control and monitoring system are not complex.

In terms of limitations, the system could potentially improve further by implementing automation steps such as automated power control based on temperature or thermal dose measurements. Furthermore, the current workflow slightly underestimates the thermal dose received by the target by not taking into account the thermal dose still being received while the target is being rapidly cooled due to its contact with air after the treatment. A solution also involving temperature measurements after heating target removal from the applicator could be considered for more accurate temperature estimation. Another solution would be to leave the heating target in the applicator until temperatures return to less than 37 °C. This would, however, increase the thermal dose received by the spheroids. Finally, it could be advantageous to consider preheating the saline solution to a temperature close to 37 °C to reduce the time the sample is exposed to temperatures under 37 °C, which might be suboptimal for the cell samples. On the other hand, in the current workflow, the samples are rapidly transferred to the applicator and the treatment can start within seconds of their removal from the incubator.

To validate the setup, we conducted two heating experiments with different target temperatures and treatment durations. The heating experiments revealed that the required temperature can be reached within a few minutes, and the target temperature can be held stable. The thermal dose accuracy was within the reproducibility uncertainty of <3% calculated from the evaluated 15 min @ 43 °C treatment. A relatively short exposure time was used for the reproducibility experiments in order to increase the relative impact of heat up and cool down time on the thermal dose. On the other hand, it should be noted that very short heat treatments with very high temperatures might lead to higher uncertainties. To also validate that the heating propagates to the target spheroids, we imaged the heated U87 MG human tumor-derived glioblastoma cell line spheroids and showed a marginal decrease in volume for a hyperthermia CEM43 dose of 30 min and a drastic decrease in volume for an ablative CEM43 dose of 493.6 min, which is in agreement with the expected direct cytotoxic effect with hyperthermic and ablative thermal doses, respectively.

In conclusion, the developed applicator is a promising tool for further studies on the biological effects of hyperthermia on cancer cells using 3D cell spheroids. This experimental device can now be employed to study biological differences in 3D cell spheroids when heating with different heating methods. We intend to use this device in a comparison with water bath heating, as well as to study the possible non-thermal biological effects of amplitude-modulated RF heating. Finally, this device can be employed in a more extended comparison between different clinically used heating techniques, including nanoparticle heating and focused ultrasound heating.

## Figures and Tables

**Table 1:** Material properties used in the EM and thermal simulations, i.e., density ρ, electrical conductivity σ, relative permittivity ε_r_, heat capacity *c*_p_, and thermal conductivity *k*.

Material	ρ (kg/m^3^)	σ (S/m)	ε_r_	*c*_p_ (J/(kg K))	*k* (W/(m K))
HDPE	950	0.00001	2.1	1900	0.50
PVC	1380	0.0001	3.0	1250	0.20
PP	920	0.0005	2.3	1800	0.15
Saline solution	994	0.2–0.8	78.2	4180	0.61
Cell medium	1050	1.63	77.2	3617	0.52

## Data Availability

The data presented in this study can be made available upon request to the corresponding author.
